# Effect of ejaculation on Serum Prostate-Specific Antigen concentration

**DOI:** 10.1590/S1677-5538.IBJU.2015.0116

**Published:** 2016

**Authors:** Fatih Tarhan, Kadir Demir, Asuman Orçun, Ozlem Cakır Madenci

**Affiliations:** 1 Department of Urology; Dr. Lütfi Kırdar Kartal Training and Research Hospital, Istanbul, Turkey; 2 Biochemistry Laboratory, Dr. Lütfi Kırdar Kartal Training and Research Hospital, Istanbul, Turkey

**Keywords:** Prostate-Specific Antigen, Ejaculation, Serum

## Abstract

**Abstract:**

Purpose:To evaluate the effect of ejaculation on serum prostate-specific antigen (PSA) concentrations in patients with lower urinary tract symptom (LUTS).

**Materials and Methods:**

Our study includes 98 men (62 study and 36 control). After three days of sexual abstinence, blood samples were drawn for the measurement of baseline PSA levels. Then the patients were told to ejaculate. One, 5, 24 and 72 hours after ejaculation, serum total (tPSA), free (fPSA) and complexed PSA (cPSA) levels were measured. Serum PSA sampling was performed at the same intervals in the control group without ejaculation.

**Results:**

The mean age in study and control groups patients were 59.03±0.99 years, 61.14±1.30 years, respectively. In the study group, changes in tPSA and fPSA levels after ejaculation were found statistically significant while changes in cPSA levels and f/tPSA ratios were not significant (p=0.016, p=0.0003, p=0.176, and p=0.173, respectively). Baseline values showed significant differences with 1st and 5th hours. No significant changes in tPSA, fPSA, cPSA levels and f/tPSA values were found in control group (p=0.223, p=0.224, p=0.444, and p=0.718, respectively). The changes in the number of patients exceeding the cutoff values after ejaculation were not statistically significant for tPSA, cPSA, and f/tPSA ratio.

**Conclusions:**

In this study, ejaculation increased tPSA and fPSA concentrations but it didn’t have a significant effect on serum cPSA levels and f/tPSA ratios. However, recent ejaculation may affect the biopsy indication at least near cut off PSA values. Further studies are needed to explain the mechanisms of alterations in the concentration of PSA.

## INTRODUCTION

Prostate-specific antigen (PSA) is a serine protease produced by epithelial cells of the prostate and it is involved in the liquefaction of seminal fluid. PSA has been widely used as a tumor marker in the screening and follow-up of prostate cancer (1). In serum, PSA exists in different molecular forms, being predominantly complexed with a1-antichymotrypsin (ACT) and a2-macroglobulin (A2M). The form of PSA which forms complexes with A2M remains undetectable because of the nature of the macroglobulin molecule. The major form which is bound to ACT is known as complexed PSA (cPSA) and the smaller unbound portion as free PSA (fPSA) cPSA and fPSA represent the main fraction of total PSA (tPSA) (2) cPSA is the dominant form in prostate cancer, whereas fPSA represents a bigger fraction in patients with benign prostatic hyperplasia.

The tPSA assay has low specificity for prostate cancer detection. Several approaches, such as those employing PSA density, PSA velocity, age-specific reference ranges, fPSA: tPSA ratio and cPSA, have been introduced to improve the diagnostic efficiency of serum PSA measurements. Similar results are obtained with cPSA compared to the fPSA: tPSA ratio, but the former process measures a single analyse instead of two, which is an economic advantage.

Since serum PSA measurement plays such a crucial role in the workup and management of prostate cancer, any urologic intervention that can cause PSA elevation has to be identified. It has been well documented that various diagnostic and therapeutic procedures causing elevations of serum PSA levels have been documented (3, 4). Also several studies have looked at the effect of ejaculation on the serum tPSA, and fPSA levels (5-16). However, no data have been available on the effect of ejaculation on the serum cPSA levels.

To examine this issue further, we conducted a prospective clinical study in order to evaluate the effects of ejaculation on serum PSA concentrations and possible contribution on clinical decision in patients with lower urinary tract symptom (LUTS).

## MATERIALS AND METHODS

Between June 2012 and May 2014, 140 male patients over 45 years of age who presented to our outpatient clinic for the first time with LUTS were screened. The International Prostate Symptom Score (IPSS), digital rectal examination, prostate volume and post void residual urine (PVR) volume measurement using suprapubic ultrasonography, uroflowmetry, urinalysis and culture were performed in all patients. Patients with a history of prostatic surgery, prostatic needle biopsy, prostate cancer, and those taking alpha-reductase inhibitors were excluded from the study. Patients with urethral catheters, urethral stricture, acute urinary tract infection, or urinary retention were also not included. A total of 42 (30%) patients (entry criteria not met by 25 patients, patient decision in 12 patients, and physician decision in 5 patients) were excluded from the study.

Remaining 98 sexually active heterosexual men (mean age 60.58±0.85 years) were included in this study. Sixty two of them masturbated and constituted the study group and 36 men who didn’t accept masturbation were taken as the control group. This study was approved by the Hospital Ethics Committee (Approval no. 15.05.2012-06). Written informed consent was obtained from all participants before enrolment.

All participating patients were instructed to abstain from ejaculation for 3 days preceding blood sampling. Blood samples were drawn by venipuncture from all men at 9-10a.m. for measurement of the baseline PSA values. Then sixty two men in the study group masturbated. The presence of semen in a specimen container confirmed the ejaculatory event. Controls experienced no ejaculation during the study period. Repeated blood samples were taken from all men at 1^st^, 5^th^, 24^th^, and 72^th^ hours after ejaculation. There was no further ejaculation during this period.

All serum samples were separated within 2 hours for analysis of the PSA forms and stored at -20ºC according to the manufacturer’s recommendations. All measurements were held in one run to exclude any between-run variations. Levels of tPSA and fPSA were measured on Immulite 2000 (Siemens, USA) and cPSA on ADVIA-Centaur (Bayer Diagnostics, Germany) analysers by chemiluminescense technology. Analytical coefficient of variation (CV) for tPSA, fPSA, and cPSA assays were 2.59%, 3.26%, and 3.0% respectively. Cut-off values for tPSA, cPSA and the fPSA: tPSA ratios were defined as ≤2.5ng/mL, ≤2.3ng/mL, and ≥0.25, respectively (17-19). Change in PSA values before and after ejaculation was given as ‘percentage of change’ (∆%) (Difference between timely measurement with basal level/basal level x 100).

Data are presented as mean±standard error of mean (SEM). Statistical calculations were performed using the paired and unpaired t-tests, ANOVA, Tukey post-test analysis, McNemar’s tests, and Pearson correlation analysis using Prizm 5.0 (GraphPad Software, USA). p<0.05 was considered significant.

## RESULTS

The mean age of study and control group were 59.03±0.99 years and 61.14±1.30 years, respectively (p=0.203). The mean±SEM values for patient age, IPSS, prostate volume, maximal urine flow rate (Qmax), PVR, and baseline PSA forms are presented in Table-1. No statistically significant differences were found for any of the parameters between two groups (Table-1).

**Table 1:** Baseline characteristics of the patients.

Variable	Study group (n= 62)	Control group (n=36)	p*
**Age (years)**	59.03±0.99	61.14±1.30	0.203
**IPSS**	12.46±0.97	14.11±1.06	0.267
**PV (mL)**	54.40±3.76	60.06±5.65	0.388
**Qmax (mL/s)**	14.80±0.58	13.23±0.89	0.122
**PVR (mL)**	33.79±5.68	40.56±6.82	0.453
**tPSA (ng/mL)**	3.46±0.54	3.52±0.52	0.937
**fPSA (ng/mL)**	0.96±0.15	0.99±0.15	0.908
**fPSA/tPSA ratio**	0.29±0.01	0.28±0.00	0.538
**cPSA (ng/mL)**	2.63±0.42	2.82±0.45	0.775

(Mean ± SEM) *Unpaired t test. (**IPSS =** International Prostate Symptom Score; **PV =** prostate volume; **Qmax**= peak flow rate; **PVR** = postvoid residual urine volume; **tPSA** = total prostate-specific antigen; **fPSA** = free PSA; **cPSA** = complexed PSA).

**Figure 1:**
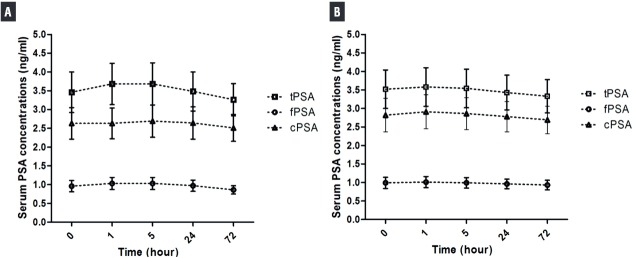
The mean values of PSA forms at baseline (zero), 1st, 5th, 24th and 72th hours’ time points of study (A) and control (B) groups. (Mean±SEM) (tPSA=total prostate-specific antigen; fPSA=free PSA; cPSA=complexed PSA).

The mean values of all PSA forms at baseline (zero), 1^st^, 5^th^, 24^th^ and 72^th^ hours’ time points of study and control groups are presented in Figure-1. In study group, comparing to baseline levels, tPSA, fPSA, and cPSA concentrations seemed to increase progressively beginning from the first hour, changes exceeding the analytical CV’s of the tests, and turn to baseline levels after 24^th^ hour. This increase of tPSA and fPSA levels were also statistically significant (p=0.016, p=0.0003, respectively), but of cPSA was not (p=0.176). In post-test analysis, tPSA and fPSA, on both 1^st^ and 5^th^hours values showed statistically significant differences from baseline values (Figure-1).

In control group, comparing to baseline levels tPSA, fPSA, and cPSA seemed to increase during the first hours and all PSA forms turned to basal levels after the 5^th^ hour. But the magnitude of these changes was within the analytical CV’s of tests. Hence none of them were statistically significant (p=0.223, p=0.224, and p=0.444, respectively).

When study and control groups were compared, a statistically significant difference was detected between ∆% 1^st^ hour and ∆% 5^th^ hour values of total and free PSA (p<0.05). However, there weren’t any significant differences between ∆% 24^th^ hour and ∆% 72^th^ hour values (p>0.05). ∆% cPSA values didn’t differ significantly from each other at any time points (p>0.05) (Table-2).

**Table 2:** The mean %∆ PSA values for study and control groups.

	%∆1^st^ hour	%∆5^th^ hour	%∆24^th^ hour	%∆ 72^th^ hour
**Study group tPSA**	10.52±1.64	7.86±1.77	1.53±1.66	1.49±1.76
**Control group tPSA**	1.69±1.08	-0.19±1.21	2.00±5.82	-3.00±2.66
p*	0.0002	0.0018	0.9238	0.1469
**Study group fPSA**	9.91±3.00	8.82±2.01	1.12±1.63	-4.06±1.71
**Control group fPSA**	1.25±1.05	-0.14±1.16	1.44±5.74	-3.22±2.64
p*	0.0338	0.0018	0.9483	0.7798
**Study group cPSA**	4.26±2.08	4.68±1.77	1.72±2.22	2.84±2.50
**Control group cPSA**	2.57±2.24	1.54±2.47	0.47±2.59	-0.91±3.17
p*	0.6000	0.2948	0.7239	0.3601

(Mean ± SEM). (tPSA = total prostate-specific antigen; fPSA = free PSA; cPSA = complexed PSA). *Unpaired t test.

The number of patients exceeding the cut-off points of tPSA, f/tPSA ratio, and cPSA before and after ejaculation is shown in Table-3. In the study and control groups, the changes in the number of patients exceeding the cut-off values after ejaculation were not statistically significant for tPSA, cPSA, and f/tPSA ratio (Table-3).

**Table 3:** Number of patients exceeding cut off points for tPSA, f/tPSA ratio, and cPSA before and after ejaculation.

	tPSA>2.5 ng/mL		fPSA/tPSA<0.25		cPSA>2.3 ng/mL	
	Study Group (n=62) n (%)	Control Group (n=36) n (%)	Study Group (n=62) n (%)	Control Group (n=36) n (%)	Study Group (n=62) n (%)	Control Group (n=36) n (%)
Before	26 (41.9)	23 (63.9)	15 (24.2)	14 (38.9)	22 (35.5)	17 (42.7)
1. hour	26 (41.9)	23 (63.9)	13 (20.9)	13 (36.1)	22 (35.5)	18 (50.0)
5. hour	27 (43.5)	23 (63.9)	11 (17.7)	14 (38.9)	22 (35.5)	16 (44.4)
24. hour	27 (43.5)	22 (61.1)	10 (16.1)	15 (41.7)	23 (37.1)	17 (42.7)
72. hour	26 (41.9)	23 (63.9)	11 (17.7)	15 (41.7)	23 (37.1)	17 (42.7)
*p	0.480 †/‡/§;1.000 ¶	0.480 †/¶; 0.617 ‡/§	0.617 †/‡; 1.000 §/¶	0.480 †/‡/§/¶	0.480 †/‡/§/¶	0.480 †/‡/§/¶

*McNemar’s test; †basal vs. 1hour; ‡basal vs. 5hour; §basal vs. 24hours; ¶ basal vs. 72hours (tPSA=total prostate-specific antigen; fPSA=free PSA; cPSA=complexed PSA)

In our study, prostate volumes of study group showed significant correlation with ∆% values of 5^th^hour for both tPSA (p=0.003, r=0.381) and fPSA (p=0.005, r=0.364). Also a significant correlation was detected between baseline PSA levels and % values of 5^th^ hour for both tPSA (p=0.0002, r=0.452) and fPSA (p=0, 0003, r=0.443). We did not detect any correlation between the patient age and ∆% values of total and free PSA.

## DISCUSSION

Although PSA is thought to be the best marker for detecting early prostate cancer, its low specificity comprises problem. In order to increase diagnostic efficiency, it’s important to know and avoid the conditions causing PSA elevations other than prostate cancer.

Ejaculation has been claimed to be one of the factors which might increase PSA values, and its effect was evaluated in some studies (5-16), but recent studies are lacking. Also, majority of these studies examined the effect of ejaculation on serum tPSA. To our knowledge, there is only one published human study about fPSA, and no study about cPSA levels. In this study about fPSA, Herschman et al. found increases in fPSA after ejaculation (12). Our study confirms their finding.

These studies investigating the effect of ejaculation on total and fPSA levels, with different conclusions, are summarized in Table-4. Researchers reported conflicting data showing increased (10-12, 16), unchanged (5, 7-9, 13-15), or decreased (6) levels of tPSA after ejaculation. However, in studies which reported PSA decreased or unchanged, sample collection time intervals after ejaculation is too long for detection of early PSA elevations we observed during first hours. Thus these studies seem to miss the detection of PSA increase. Some of the reported differences may have been due to population biases owing to the small groups of patients studied (6, 8, 11, 12). Five of the studies included control group (6, 7, 9, 11, 14). Only six of them were done with men over 45 years of age that is proposed for prostate cancer screening (7, 9, 10, 12, 15, 16). Also, the evaluation of ejaculation, sample timing, and PSA measurement methods vary importantly among studies. This variability causes difficulties in interpretation. Our study includes control group, tPSA, fPSA and cPSA as parameters, and regular sampling times for both groups after ejaculation as different from previous studies. Additionally, our study is the first to report the effects of ejaculation on serum cPSA levels in human beings (Table-4).

**Table 4:** Summary of previous reports of the effect of ejaculation on the forms of PSA.

Reference	n	PSA	Time of Blood Sampling	Findings
Glenski et al. (5)	30 (study)	Total	Before and 17.5-28.8 hour after ejaculation.	Ejaculation has an insignificant effect on serum PSA values.
Simak et al. (6)	18 (study) 3 (control)	Total	Before and 1, 7 days after ejaculation.	PSA decreased after ejaculation
McAleer et al. (7)	35 (study) 81 (control)	Total	Before and mean 14.6 hour after ejaculation.	Ejaculation has an insignificant effect on serum PSA values.
Kirkali et al. (8)	18 (study)	Total	Before and 5 consecutive days after ejaculation.	Ejaculation has an insignificant effect on serum PSA values.
Netto et al. (9)	40 (study) 10 (control)	Total	Before and 1, 7. days after ejaculation.	Ejaculation has an insignificant effect on serum PSA values.
Tchetgen et al. (10)	64 (study)	Total	Before and 1, 6, 24, 48 hours after ejaculation.	PSA significantly increased after ejaculation up to 48 hours.
Zisman et al. (11)	18 (study) 16 (control)	Total	Before and 1 hour after ejaculation.	PSA significantly increased after ejaculation.
Herschman et al. (12)	20 (study)	Total and free	Before and 1, 6, 24 hours after ejaculation.	Both tPSA and fPSA significantly increased after ejaculation up to24 hours.
Heindreich et al. (13)	100 (study)	Total	Before and 1, 24 hours after ejaculation.	Ejaculation has an insignificant effect on serum PSA values.
Yavasçaoglu et al. (14)	25 (study) 20 (control)	Total	Before and 1, 5 days after ejaculation.	Ejaculation has an insignificant effect on serum PSA values.
Stenner et al. (15)	89 (study)	Total	Before and mean10.18 hour after ejaculation.	Ejaculation has an insignificant effect on serum PSA values.
Rajaei et al. (16)	60 (study)	Total	Before and 1, 24 hours after ejaculation.	PSA significantly increased within 1 hour after ejaculation.

In the study group, the serum levels of all forms of PSA increased at 1^st^ hour after ejaculation and returned to baseline at 24^th^ hours. Ejaculation induced a more pronounced increase in tPSA (10.5%) than in fPSA (9.9%) and in cPSA (4.3%). These increases were statistically significant for tPSA and fPSA. A statistically significant increase in the control group was not detected in any form of PSA. In this case we can say that serum PSA levels increase after ejaculation. Indeed, some studies have confirmed this result (10-12, 16).

In our study, we found a statistically significant increase of tPSA and fPSA after ejaculation in the study group; but not in the control group. In contrast to other PSA forms, less and insignificant cPSA elevations were observed after ejaculation. Changes in the number of patients exceeding the cut-off values after ejaculation were also not statistically significant for tPSA, cPSA, and f/tPSA ratio. This situation, although not statistically significant, should be expected to be of clinical significance in individual patients close to the threshold levels.

We consider that increase in tPSA levels after ejaculation may be due to the increase in fPSA levels. The site of cPSA formation is controversial; it may be in the prostate gland, or in the circulation (20). In vitro studies sustain that complex formation of fPSA with ACT lasts longer than that with α2 macroglobulin (21). Insignificant variation of cPSA levels after ejaculation may be explained with kinetics of serum PSA isoforms.

The effect of ejaculation on serum PSA forms might be different in various prostatic diseases as BPH, prostate carcinoma, and chronic prostatitis. We did not investigate this this issue in this study. This may be the limitation of our study.

The prostate volume and baseline PSA concentration were found as factors affecting serum PSA levels after ejaculation. Elevation of PSA levels after ejaculation is greater in patients with a larger prostate volume and higher baseline PSA values. However, the change in serum PSA after ejaculation was independent of the patient’s age. Tchetgen et al. found that declining to basal levels after ejaculation take longer in older patients and in patients with higher baseline PSA levels (10). Benign prostatic hyperplasia causing ductal obstruction, acinar dilatation, and secretion retention besides membrane weakness and thus disturbed barrier permeability can cause PSA leakage during ejaculation (10). Additionally, pelvic muscle and periprostatic contractions can cause PSA passage into circulation (15). Studies showing PSA increases in elderly or patients with BPH supports the mechanism of hyperplastic tissue compression and barrier disturbance.

## CONCLUSIONS

In this study, ejaculation increased tPSA and fPSA concentrations but it didn’t have a significant effect on serum cPSA levels. However, increase of cPSA and decrease f/tPSA ratio after ejaculation may cause some patients to exceed corresponding cut-off levels, although not statistically significant. Therefore, sexual abstinence should be advised for 24 hours before any PSA measurement to avoid nonspecific interpretations of serum PSA levels. Further research is needed to explain the mechanism of alterations in the concentrations of PSA.
